# Supplementary material for: A tuft cell - ILC2 signaling circuit provides therapeutic targets to inhibit gastric metaplasia and tumor development
Source: Nat Commun. 2023 Oct 28;14:6872. doi: 10.1038/s41467-023-42215-4 (PMC10613282; doi:10.1038/s41467-023-42215-4)
Supplement: Supplementary file 1 — Supplementary Information [file 41467_2023_42215_MOESM1_ESM.pdf]

Supplementary Information for

A tuft cell - ILC2 signaling circuit provides therapeutic targets to inhibit gastric metaplasia and tumor development

Ryan N O'Keefe, Annalisa LE Carli, David Baloyan, David Chisanga, Wei Shi, Shoukat Afshar-Sterle, Moritz F Eissmann, Ashleigh R Poh, Bhupinder Pal, Cyril Seillet, Richard M Locksley, Matthias Ernst[&], Michael Buchert[&,*]

*Corresponding author. Email: Michael.buchert@onjcri.org.au

This PDF file includes:

Supplementary Figure 1 to 8
Supplementary Tables 1 to 3

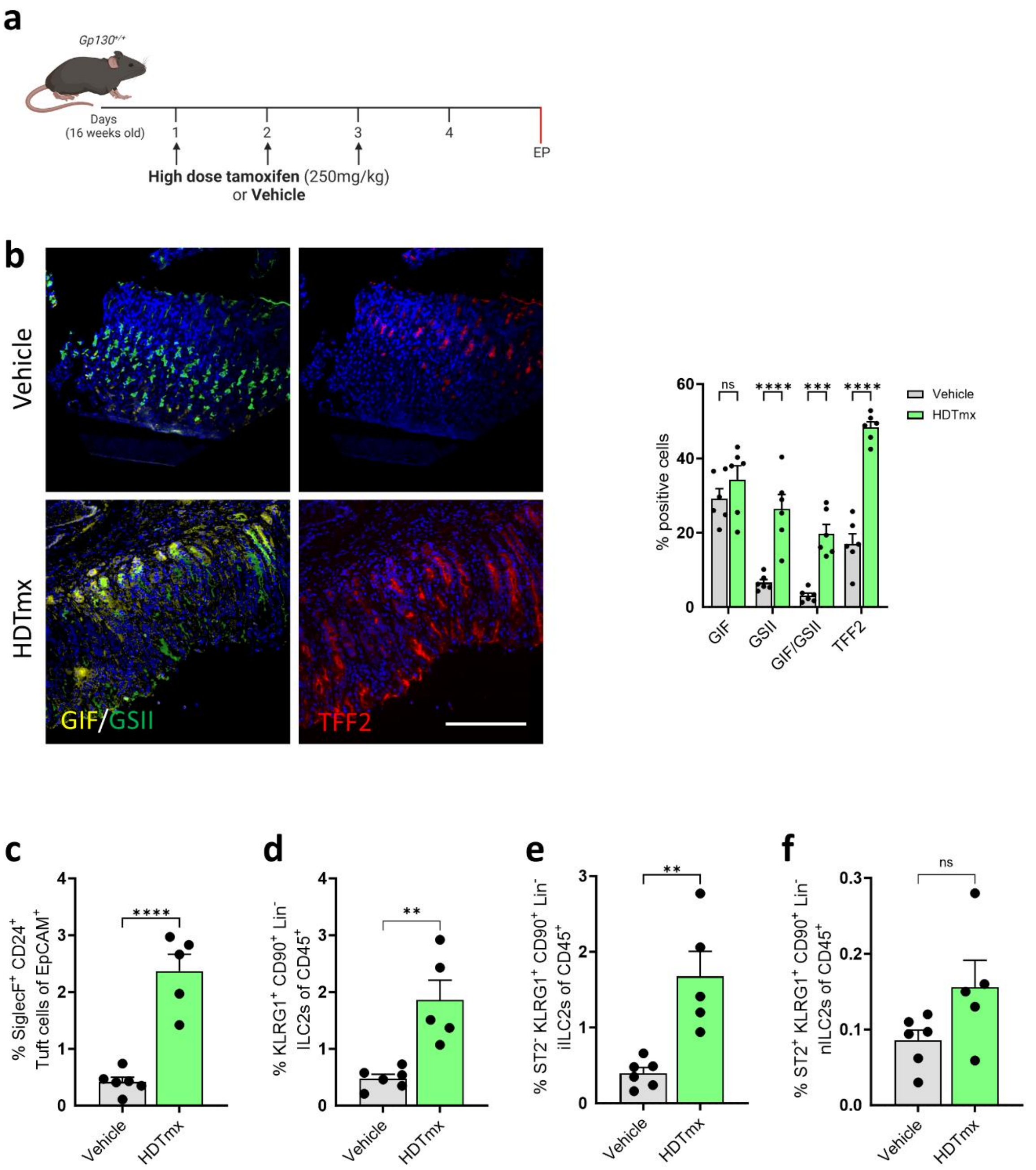
a
Gp130+/+
Days
(16 weeks old)
1
2
3
4
EP
High dose tamoxifen (250mg/kg)
or Vehicle
b
Vehicle
HDTmx
GIF/GSII
TFF2
% positive cells
ns
****
***
****
Vehicle
HDTmx
GIF
GSII
GIF/GSII
TFF2
c
% SiglecF+ CD24+ Tuft cells of EpCAM+
****
Vehicle
HDTmx
d
% KLRG1+ CD90+ Lin- ILC2s of CD45+
**
Vehicle
HDTmx
e
% ST2- KLRG1+ CD90+ Lin- iILC2s of CD45+
**
Vehicle
HDTmx
f
% ST2+ KLRG1+ CD90+ Lin- nILC2s of CD45+
ns
Vehicle
HDTmx

Supplementary Figure 1. Tuft cells and ILC2s are increased during Spasmolytic polypeptide-expressing metaplasia (SPEM), a precursor to gastric cancer

(**a**) Schematic of experimental SPEM model. 16-week-old *gp130*$^{+/+}$ mice were treated with either a vehicle control or high dose of tamoxifen (HDTmx, 250mg/kg) once daily for 3 consecutive days to induce loss of parietal cells and gastric spasmolytic polypeptide-expressing metaplasia (SPEM). EP = endpoint. Created with BioRender.com.

(**b**) Representative immunofluorescent images and quantification of GIF/GSII-lectin and TFF2 positively-stained gastric mucosa of mice, treated as described in Supplementary Figure 1a. Scale bar = 200μm. N = 6 and 6 respectively.

(**c**) Flow-cytometry quantification of SiglecF^{+}CD24^{+}EpCAM^{+} tuft cells in stomachs of mice, treated as described in Supplementary Figure 1a. N = 6 and 5 respectively.

(**d**) Flow-cytometry quantification of total ILC2s as KLRG1^{+}CD90.2^{+}Lineage^{-}CD45^{+} in stomachs of mice, treated as described in Supplementary Figure 1a. N = 6 and 5 respectively.

(**e**) Flow-cytometry quantification of iILC2s as ST2^{-}KLRG1^{+}CD90.2^{+}Lineage^{-} CD45^{+} in stomachs of mice, treated as described in Supplementary Figure 1a. N = 6 and 5 respectively.

(**f**) Flow-cytometry quantification of nILC2s as ST2^{+}KLRG1^{+}CD90.2^{+}Lineage^{-}CD45^{+} in stomachs of mice, treated as described in Supplementary Figure 1a. N = 6 and 5 respectively.

Data represents mean ± SEM, p values from two-sided Student's t-test ** $p < 0.01$, *** $p< 0.001$, **** $p< 0.0001$, ns - not significant. Each symbol represents an individual mouse. Data is pooled from two independent experiments. Source data and exact p values are provided as a Source Data file.

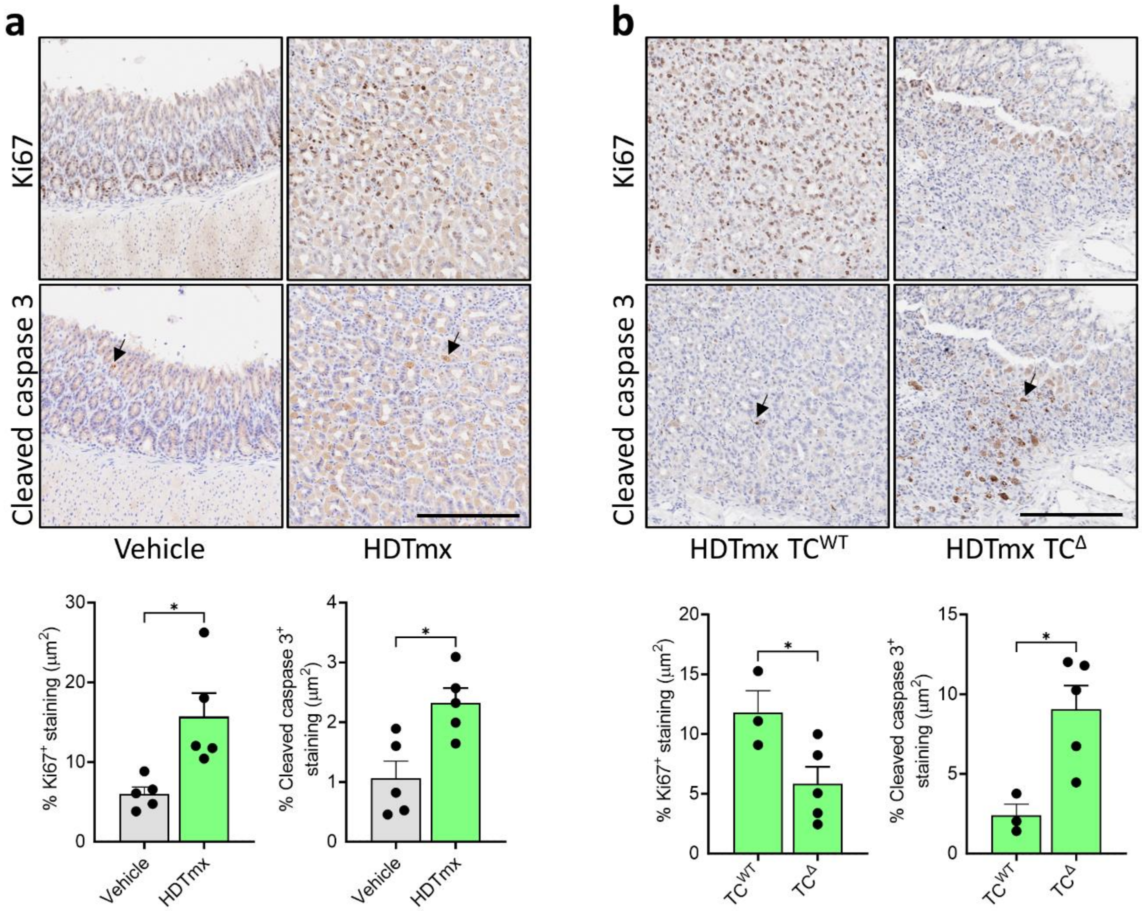

Supplementary Figure 2. In SPEM, proliferation is decreased and cell death is increased following tuft cell loss

(**a**) Representative IHC images and quantification of Ki67 and Cleaved caspase 3 stained gastric mucosa of vehicle and HDTmx treated WT mice. Scale bar = 300µm. Arrows indicate positive staining. N = 5 and 5 respectively.

(**b**) Representative IHC images and quantification of Ki67 and Cleaved caspase 3 stained gastric mucosa of HDTmx treated TC^{WT} and TC^{Δ} mice. Scale bar = 300µm. Arrows indicate positive staining. N = 3 and 5 respectively.

Data represents mean ± SEM, p values from two-sided Student's t-test * $p < 0.05$. Each symbol represents an individual mouse. Source data and exact p values are provided as a Source Data file.

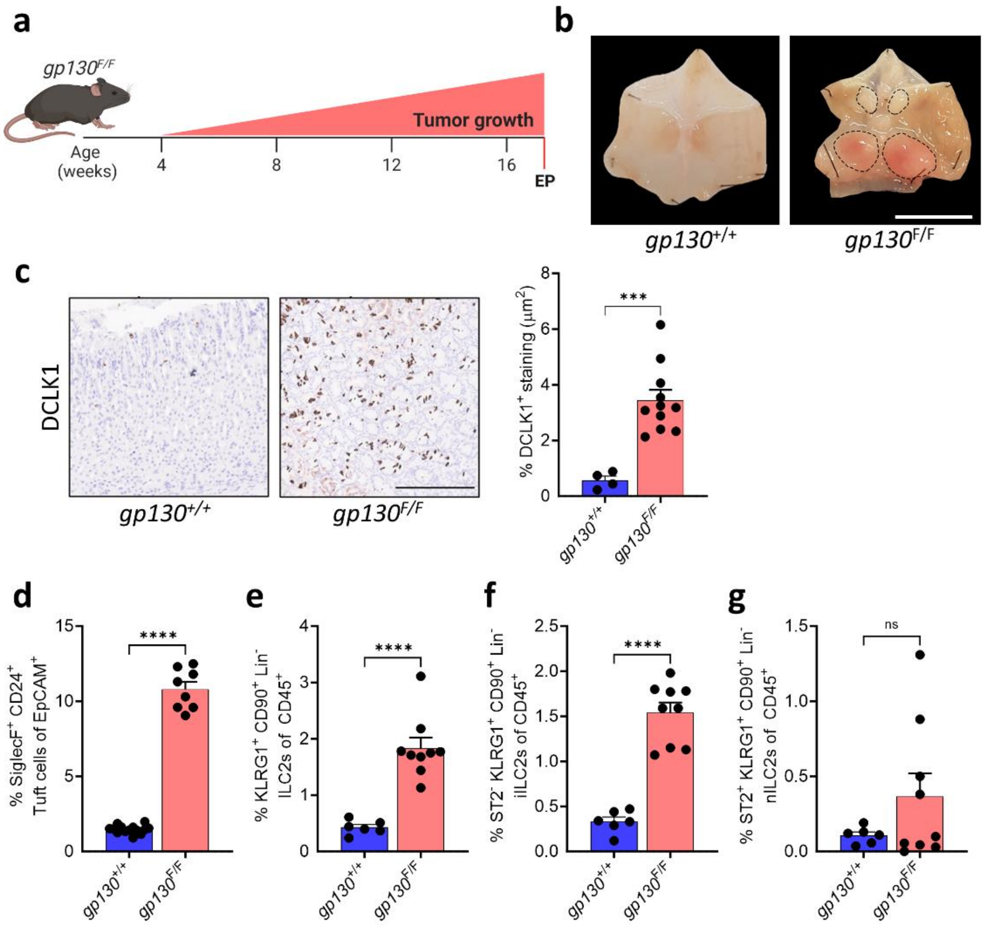

Supplementary Figure 3. Tuft cells and ILC2s are increased in during gastric tumor development

(**a**) Schematic of the *gp130*$^{F/F}$ mouse model of gastric cancer, which spontaneously develops gastric adenomas from 4 weeks of age. EP = endpoint.

(**b**) Representative IHC images of *gp130*$^{+/+}$ and *gp130*$^{F/F}$ stomachs at 17 weeks of age. Dotted circles indicate tumors, scale bar = 8mm.

(**c**) IHC quantification of tuft cells (DCLK1^{+}) in *gp130*$^{+/+}$ and *gp130*$^{F/F}$ mice. Scale bar = 300mm. N = 4 and 11 respectively.

(**d**) Flow-cytometry quantification of tuft cells as SiglecF^{+}CD24^{+}EpCAM^{+} cells in stomachs of *gp130*$^{+/+}$ and *gp130*$^{F/F}$ mice. N = 14 and 8 respectively.

(**e**) Flow-cytometry quantification of total ILC2s as KLRG1^{+}CD90.2^{+}Lineage^{-}CD45^{+} cells in stomachs of *gp130*$^{+/+}$ and *gp130*$^{F/F}$ mice. N = 6 and 9 respectively.

(**f**) Flow-cytometry quantification of iILC2s as ST2^{-}KLRG1^{+}CD90.2^{+}Lineage^{-} CD45^{+} cells in stomachs of *gp130*$^{+/+}$ and *gp130*$^{F/F}$ mice. N = 6 and 9 respectively.

(**g**) Flow-cytometry quantification of nILC2s as ST2^{+}KLRG1^{+}CD90.2^{+}Lineage^{-}CD45^{+} cells in stomachs of *gp130*$^{+/+}$ and *gp130*$^{F/F}$ mice. N = 6 and 9 respectively.

Data represents mean ± SEM, p values from two-sided Student's t-test *** $p < 0.001$, **** $p < 0.0001$, ns - not significant. Each symbol represents an individual mouse. Data is pooled from two independent experiments. Source data and exact p values are provided as a Source Data file.

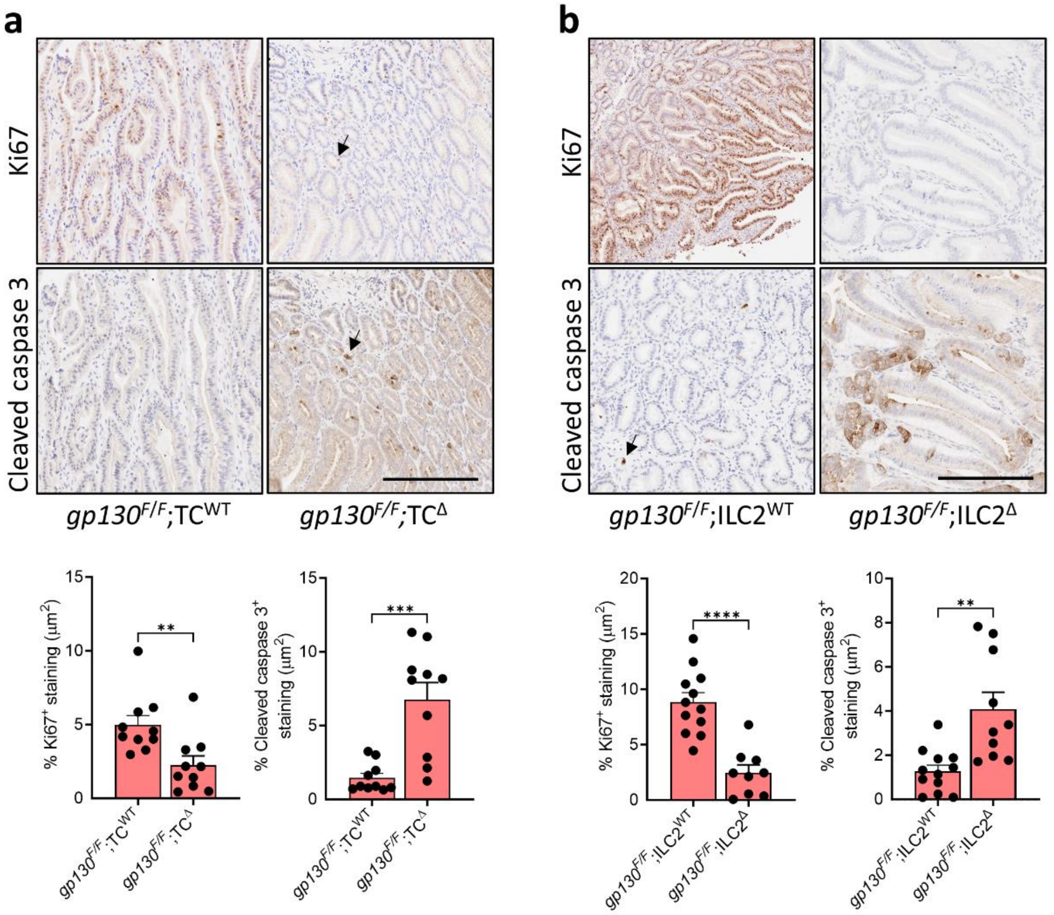

Supplementary Figure 4. In gastric tumors, proliferation is decreased and cell death is increased following loss of tuft cell and ILC2s

(**a**) Representative images and quantification of Ki67 and Cleaved caspase 3 stained gastric mucosa of *gp130*$^{F/F}$;TC^{WT} and *gp130*$^{F/F}$;TC$^{\Delta}$ mice. Scale bar = 300µm. Arrows indicate positive staining. N = 10 and 10 respectively.

(**b**) Representative images and quantification of Ki67 and Cleaved caspase 3 stained gastric mucosa of *gp130*$^{F/F}$;ILC2^{WT} and *gp130*$^{F/F}$;ILC2$^{\Delta}$ mice. Scale bar = 300µm. Arrows indicate positive staining. N = 12 and 9 respectively.

Data represents mean ± SEM, p values from two-sided Student's t-test ** $p < 0.01$, *** $p < 0.001$, **** $p < 0.0001$. Each symbol represents an individual mouse. Data is pooled from two independent experiments. Source data and exact p values are provided as a Source Data file.

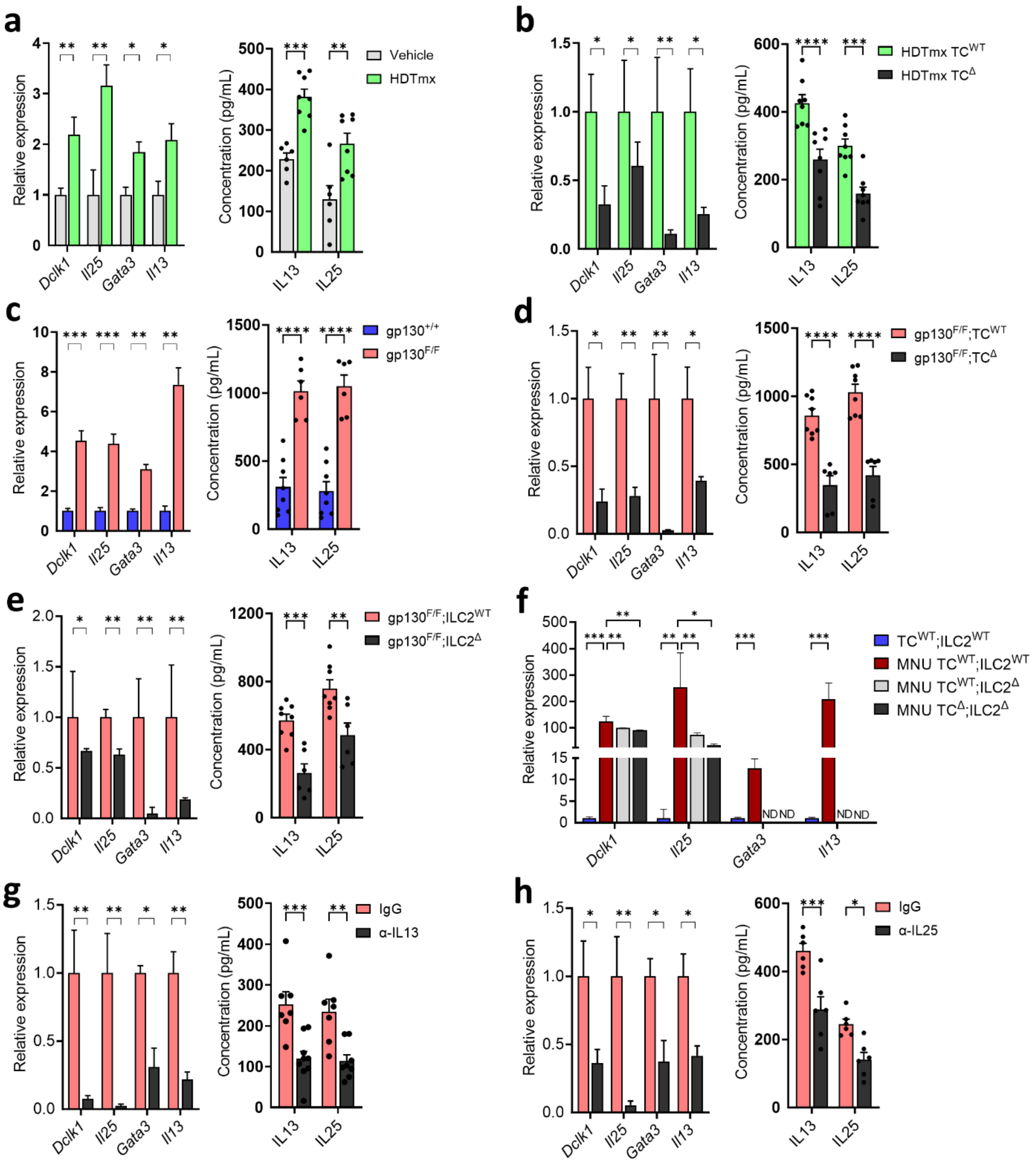
a
Relative expression
Dclk1
Il25
Gata3
Il13
Concentration (pg/mL)
IL13
IL25
Vehicle
HDTmx
b
HDTmx TC^WT
HDTmx TC^Δ
c
gp130^+/+
gp130^F/F
d
gp130^F/F;TC^WT
gp130^F/F;TC^Δ
e
gp130^F/F;ILC2^WT
gp130^F/F;ILC2^Δ
f
TC^WT;ILC2^WT
MNU TC^WT;ILC2^WT
MNU TC^WT;ILC2^Δ
MNU TC^Δ;ILC2^Δ
ND
g
IgG
α-IL13
h
IgG
α-IL25

Supplementary Figure 5. Tuft cell and ILC2 markers and cytokines are increased during early gastric metaplasia and adenoma development

Gene and protein expression analysis for tuft cell-specific (*Dclk1*, *Il25)*, and ILC2-specific (*Gata3*,*Il13)* markers in stomachs of:

(**a**) vehicle treated and HDTmx treated mice (gene expression N = 6 and 6, protein N = 6 and 8 respectively).

(**b**) HDTmx treated TC^{WT} and TC^{Δ} mice (gene expression N = 10 and 10, protein N = 8 and 8 respectively).

(**c**) 17-week-old *gp130*$^{+/+}$ and *gp130*$^{F/F}$ mice (gene expression N = 6 and 6, protein N = 6 and 8 respectively).

(**d**) Age matched TC^{WT};$ILC2^{WT}$, as well as MNU treated TC^{WT};$ILC2^{WT}$, TC^{Δ};$ILC2^{\Delta}$ and TC^{WT};$ILC2^{\Delta}$ mice (gene expression N = 6 and 6, protein N = 8 and 6 respectively).

(**e**) 17-week-old LDTmx treated *gp130*$^{F/F}$;TC^{WT} and *gp130*$^{F/F}$;TC^{Δ} mice (gene expression N = 6 and 8, protein N = 8 and 6 respectively).

(**f**) 17-week-old *gp130*$^{F/F}$;$ILC2^{WT}$ and *gp130*$^{F/F}$;$ILC2^{\Delta}$ mice (N = 9, 10, 11 and 4 respectively).

(**g**) IgG and α-IL13 treated *gp130*$^{F/F}$ mice (gene expression N = 5 and 5, protein N = 7 and 8 respectively).

(**h**) IgG and α-IL25 treated *gp130*$^{F/F}$ mice (gene expression N = 5 and 5, protein N = 6 and 6 respectively).

Data represents mean ± SEM, p values from two-sided Student's t-test or one-way ANOVA and Tukey's multiple comparisons tests * $p < 0.05$, ** $p < 0.01$, *** $p < 0.001$, ns - not significant. ND – not detected. Data is pooled from two independent experiments for each panel. Source data and exact p values are provided as a Source Data file.

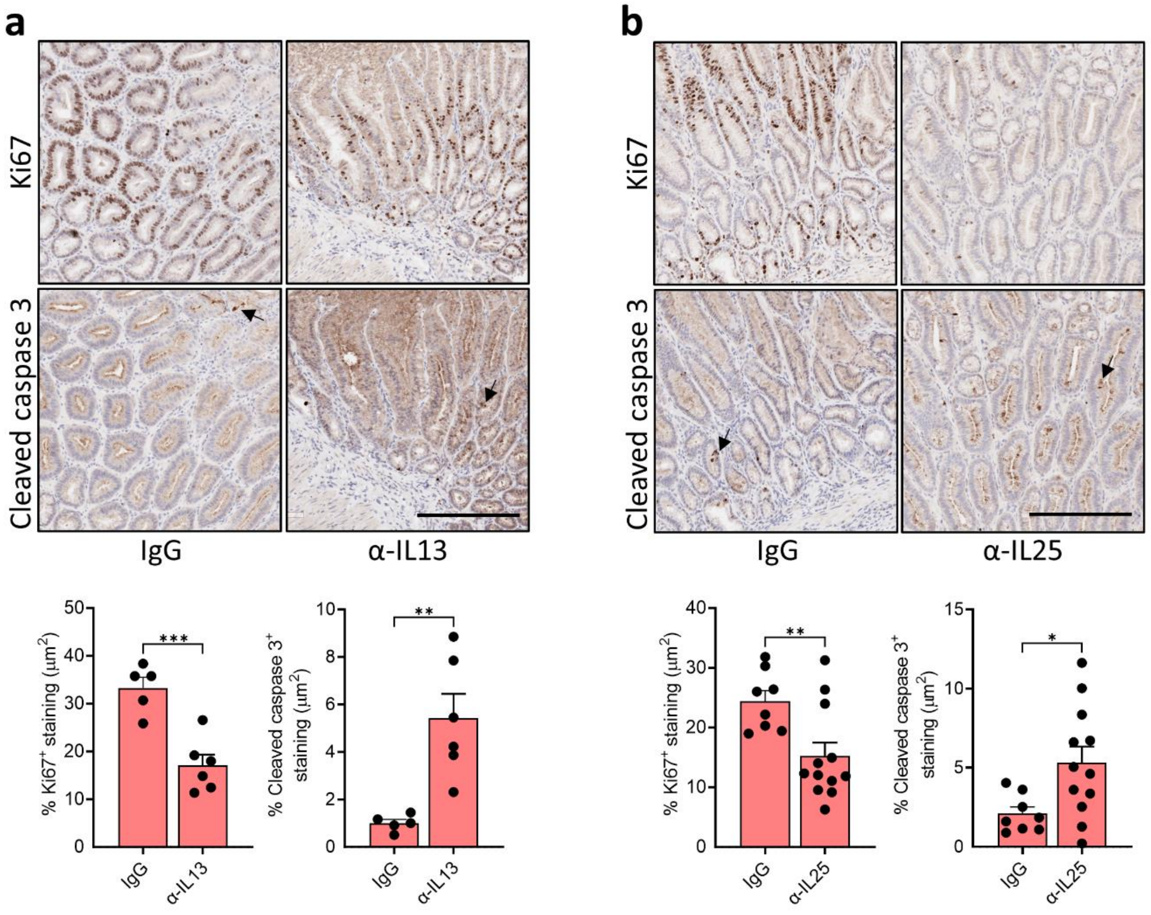

Supplementary Figure 6. In gastric tumors, proliferation is decreased, and cell death is increased following α-IL13 and α-IL25 treatment

(**a**) Representative IHC images and quantification of Ki67 and Cleaved caspase 3 stained gastric mucosa of IgG and α-IL13 treated *gp130*$^{F/F}$ mice. Scale bar = 300μm. Arrows indicate positive staining. N = 5 and 6 respectively.

(**b**) Representative images and quantification of Ki67 and Cleaved caspase 3 stained gastric mucosa of IgG and α-IL25 treated *gp130*$^{F/F}$ mice. Scale bar = 300μm. Arrows indicate positive staining. N = 8 and 12 respectively.

Data represents mean ± SEM, p values from two-sided Student's t-test * p <0.05, ** p < 0.01, *** p< 0.001. Each symbol represents an individual mouse. Data is pooled from two independent experiments. Source data and exact p values are provided as a Source Data file.

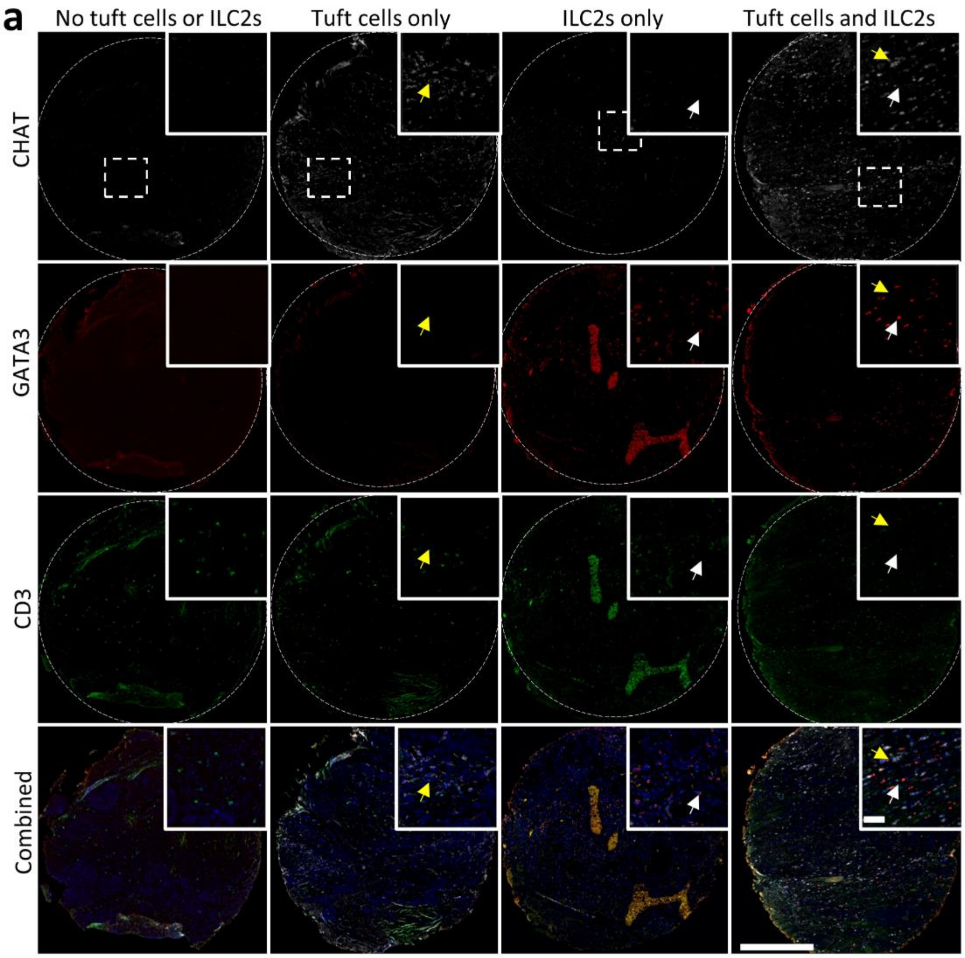

Supplementary Figure 7. Tuft cells and ILC2 are involved in human GC

(**a**) Representative Opal-IF stains for $ChAT^+$ tuft cells and $CD3^-GATA3^+$ ILC2s in human intestinal-type GC tissue microarrays, n=67 patients. Scale bar = 500µm for low magnification and = 100µm for high magnification (dotted insets). Arrows indicate positive staining.

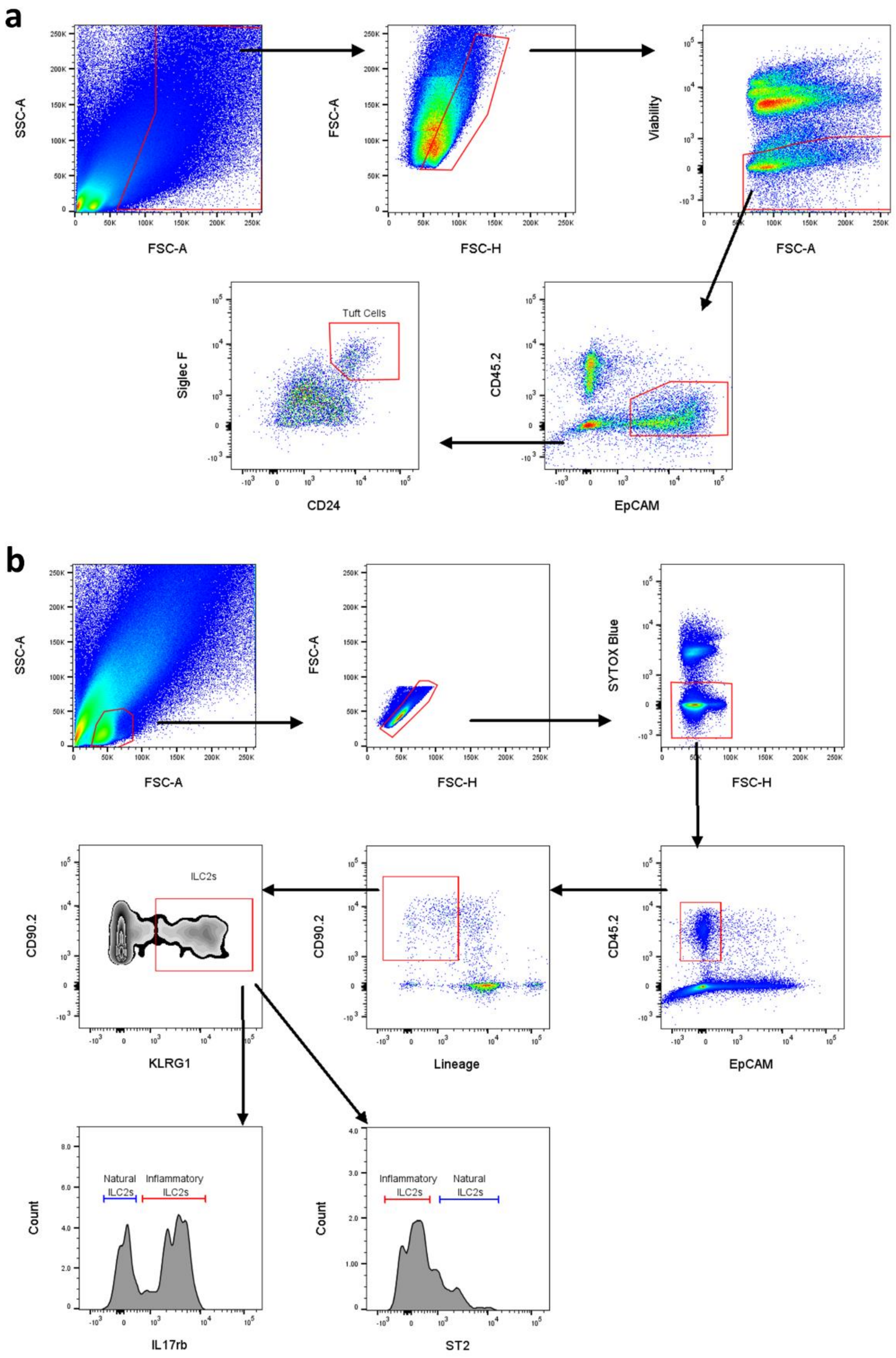

Supplementary Figure 8. Gating strategy for tuft cells and ILC2s

(**a**) Gating strategy used to identify and sort murine gastric tuft cells.
(**b**) Gating strategy used to sort ILC2s, as well as identify nILC2s and iILC2s subpopulations from mouse stomachs.

Supplementary Table 1. Oligonucleotide sequences for SYBR Green qPCR

Gene	Forward primer	Reverse primer
18S	GTAACCCGTTGAACCCCATT	CCATCCAATCGGTAGTAGCG
Dclk1	TTCAACACAGGCCCCAAG	TATCAAGAGCGGTGGTTGC
Gapdh	AAGAGGGATGCTGCCCTTA	TTTTGTCTACGGGACGAGGA
Gata3	TCGGCCATTCGTACATGGAA	GAGAGCCGTGGTGGATGGAC
Il13	CCTCTGACCCTTAAGGAGCTTAT	CGTTGCACAGGGGAGTCT
Il13ra1	TCACTTTGATGACCAACAGGAT	CAGGGGTAATTCCTCTTTACGA
Il17rb	GGACAGCCCTTCTTTGTCTG	TGCTTTTTATATTTCATTACGTGGTT
Il25	ACAGGGACTTGAATCGGGTC	TGGTAAAGTGGGACGGAGTTG

Supplementary Table 2. Primary antibodies for paraffin immunohistochemistry (IHC-P), Opal staining or paraffin immunofluorescence (IF)

Antibody	Reference	Manufacturer	Concentration
DCLK1	ab31704	Abcam	1/1000
GATA3	SC268	Santa Cruz Biotechnology	1/100
CD3	MA5-14524	Invitrogen	1/150
ChAT	AB144P	Millipore Sigma	1/50
TFF2	Pa5-80111	Invitrogen	1/500
H^+/K^+ ATPase	Ab176992	Abcam	1/500
Ki67	IHC-00375	Bethyl Laboratories	1/200
Cleaved caspase 3	#9661S	Cell Signaling	1/500
GS-II lectin	L21415	Invitrogen	1/1000
Gastric intrinsic factor (GIF)	A6914	ABclonal	1/150

Supplementary Table 3. Conjugated antibodies for flow cytometry.

Antibody	Reference	Manufacturer	Conjugate	Concentration
CD19	25-0193-81	Invitrogen	PEVio770	1/200
CD11c	25-0114-87	Invitrogen	PEVio770	1/200
CD11b	101216	BioLegend	PEVio770	1/200
CD3e	25-0031-82	Invitrogen	PEVio770	1/200
CD90.2	130-102-345	MACS	VioBlue	1/100
KLRG1	138407	BioLegend	PE	1/200
NK1.1	25-5941-81	Invitrogen	PEVio770	1/200
CD24	130-102-733	MACS	APC	1/50
SiglecF	562757	BD Bioscience	PE-CF594	1/200
CD45.2	103116	BioLegend	APC-Cy7	1/200
EpCAM	11-5791-82	Invitrogen	FITC	1/200
ST2	46-9335-82	Invitrogen	PerCP-eF710	1/200
LY6G	560601	BD Bioscience	PEVio770	1/200
FC Block CD16/CD32	14-0161-86	Invitrogen		1/100
Sytox Blue	S11348	Invitrogen		1/500
Fixable Viability Dye	65-0866-14	eBioscience	eF506	1/1000
IgG2aϰ	#400512	BioLegend	APC	1:200
IgG2aϰ	#400230	BioLegend	APC-CY7	1:200
IgG2aϰ	#400208	BioLegend	FITC	1:200
IgG2aϰ	#400908	BioLegend	PE	1:200
IgG2aϰ	#400522	BioLegend	PeCY7	1:200
