# Supplementary material for: A tuft cell - ILC2 signaling circuit provides therapeutic targets to inhibit gastric metaplasia and tumor development
Source: Nat Commun. 2023 Oct 28;14:6872. doi: 10.1038/s41467-023-42215-4 (PMC10613282; doi:10.1038/s41467-023-42215-4)
Supplement: Supplementary file 2 — Reporting Summary [file 41467_2023_42215_MOESM2_ESM.pdf]

nature portfolio

Corresponding author(s): Michael Buchert

Last updated by author(s): 2023/09/28

Reporting Summary

Nature Portfolio wishes to improve the reproducibility of the work that we publish. This form provides structure for consistency and transparency in reporting. For further information on Nature Portfolio policies, see our Editorial Policies and the Editorial Policy Checklist.

Statistics

For all statistical analyses, confirm that the following items are present in the figure legend, table legend, main text, or Methods section.

n/a	Confirmed	
☐	☒	The exact sample size (*n*) for each experimental group/condition, given as a discrete number and unit of measurement
☐	☒	A statement on whether measurements were taken from distinct samples or whether the same sample was measured repeatedly
☐	☒	The statistical test(s) used AND whether they are one- or two-sided<br>*Only common tests should be described solely by name; describe more complex techniques in the Methods section.*
☒	☐	A description of all covariates tested
☐	☒	A description of any assumptions or corrections, such as tests of normality and adjustment for multiple comparisons
☐	☒	A full description of the statistical parameters including central tendency (e.g. means) or other basic estimates (e.g. regression coefficient) AND variation (e.g. standard deviation) or associated estimates of uncertainty (e.g. confidence intervals)
☐	☒	For null hypothesis testing, the test statistic (e.g. *F*, *t*, *r*) with confidence intervals, effect sizes, degrees of freedom and *P* value noted<br>*Give P values as exact values whenever suitable.*
☒	☐	For Bayesian analysis, information on the choice of priors and Markov chain Monte Carlo settings
☒	☐	For hierarchical and complex designs, identification of the appropriate level for tests and full reporting of outcomes
☒	☐	Estimates of effect sizes (e.g. Cohen's *d*, Pearson's *r*), indicating how they were calculated

Our web collection on statistics for biologists contains articles on many of the points above.

Software and code

Policy information about availability of computer code

Data collection	Illumina Nextseq 500, Viia7 Real-Time PCR System, Vectra imaging system, Aria III cell sorter, BD FACS Canto, SPECTROstar Nano (BMGLABTECH)
Data analysis	Prism 10 software, Rsubread (v2.5.0), Microsoft Excel (version 2306), InForm (Perkin Elmer, version 2.5), Halo (Indica Labs, version 3.5), ImageJ (FiJi, version 1.53c), SingleR (version 4.3), Rsubread (version 2.5.0), Seurat (version 5), MARS Data Analysis Software (BMG LABTECH, version 4.01)

For manuscripts utilizing custom algorithms or software that are central to the research but not yet described in published literature, software must be made available to editors and reviewers. We strongly encourage code deposition in a community repository (e.g. GitHub). See the Nature Portfolio guidelines for submitting code & software for further information.

Data

Policy information about availability of data

All manuscripts must include a data availability statement. This statement should provide the following information, where applicable:

- Accession codes, unique identifiers, or web links for publicly available datasets
- A description of any restrictions on data availability
- For clinical datasets or third party data, please ensure that the statement adheres to our policy

The Single cell sequencing data generated in this study have been deposited in the Gene Expression Omnibus database under accession code GSE217498 [https://www.ncbi.nlm.nih.gov/geo/query/acc.cgi?acc=GSE217498]. The publicly available data used in this study are available in the Kaplan-Meier Plotter database [https://kmplot.com/analysis/index.php?p=service&cancer=gastric] that uses the following datasets: GSE14210, GSE15459, GSE22377, GSE29272, GSE51105 and GSE62254. The remaining data are available within the Article, Supplementary Information or Source Data file.

Research involving human participants, their data, or biological material

Policy information about studies with human participants or human data. See also policy information about sex, gender (identity/presentation), and sexual orientation and race, ethnicity and racism.

Reporting on sex and gender	Sex or gender was not considered in the study design.
Reporting on race, ethnicity, or other socially relevant groupings	Race and ethnicity were not considered in the study design.
Population characteristics	All samples were taken from patients identified as having intestinal type gastric cancer. Age and Sex were not considered when selecting for these patients.
Recruitment	We obtained paraffin-embedded samples of primary gastric adenocarcinomas (prepared as Tissue Microarray, TMA) from the Department of Pathology, Austin Hospital. All patients provided their informed consent.
Ethics oversight	All samples were collected with the patients' written informed consent and approval from theAustin Health ethics committee (HREC/15/Austin/359).

Note that full information on the approval of the study protocol must also be provided in the manuscript.

Field-specific reporting

Please select the one below that is the best fit for your research. If you are not sure, read the appropriate sections before making your selection.

☒ Life sciences ☐ Behavioural & social sciences ☐ Ecological, evolutionary & environmental sciences

For a reference copy of the document with all sections, see nature.com/documents/nr-reporting-summary-flat.pdf

Life sciences study design

All studies must disclose on these points even when the disclosure is negative.

Sample size	G*Power3 was used to calculate minimal required sample size for all experiments based on a difference between means of 40% and a SD of 20% (unpaired, two-tailed t-test; "=0.05, Power=0.95)
Data exclusions	No data was excluded
Replication	All experiments were performed at least twice to ensure data could be replicated
Randomization	All mice were randomly selected from a pool or the desired genotype. These mice were then recorded as belonging to a group, but housed as a mixed cohort to reduce variables. For all other experiments, samples were randomly allocated to groups from a pool of available samples.
Blinding	Mice were identified by a number that did not correspond to their treatment group, with all data collected prior to knowing the group each mouse belonged to. Analyses was performed while the investigators were blinded to the groups, with groups only being identified once all analysis was complete. For other experiments, blinding occurred during data collection and analysis, with group identification being performed as the final step.

Reporting for specific materials, systems and methods

We require information from authors about some types of materials, experimental systems and methods used in many studies. Here, indicate whether each material, system or method listed is relevant to your study. If you are not sure if a list item applies to your research, read the appropriate section before selecting a response.

Materials & experimental systems

n/a	Involved in the study
☐	☒ Antibodies
☒	☐ Eukaryotic cell lines
☒	☐ Palaeontology and archaeology
☐	☒ Animals and other organisms
☒	☐ Clinical data
☒	☐ Dual use research of concern
☒	☐ Plants

Methods

n/a	Involved in the study
☒	☐ ChIP-seq
☐	☒ Flow cytometry
☒	☐ MRI-based neuroimaging

Antibodies

Antibodies used	DCLK1 (Abcam, ab31704), GATA3 (Santa Cruz Biotechnology, SC268), CD3 (Invitrogen,MA5-14524), ChAT (Millipore Sigma, AB144P), TFF2 (Invitrogen, Pa5-80111), H+/K+ ATPase (Abcam, Ab176992), Ki67 (Bethyl Laboratories, IHC-00375), Cleaved caspase 3 (Cell Signaling, #9661S), GS-II lectin (Invitrogen, L21415), Gastric intrinsic factor (GIF) (ABclona, A6914), CD19 (Invitrogen, 25-0193-81), CD11c (Invitrogen, 25-0114-87), CD11b (BioLegend, 101216), CD3e (Invitrogen, 25-0031-82), CD90.2 (MACS, 130-102-345), KLRG1 (BioLegend, 138407), NK1.1 (Invitrogen, 25-5941-81), CD24 (MACS, 130-102-733), SiglecF (BD Bioscience, 562757), CD45.2 (BioLegend, 103116), EpCAM (Invitrogen, 11-5791-82), ST2 (Invitrogen, 46-9335-82), LY6G (BD Bioscience, 560601), FC Block CD16/CD32 (Invitrogen, 14-0161-86), Sytox Blue (Invitrogen, S11348), Fixable Viability Dye (eBioscience, 65-0866-14), IgG2a# (BioLegend, #400512), IgG2a# (BioLegend, #400230), IgG2a# (BioLegend, #400208), IgG2a# (BioLegend, #400908), IgG2a# (BioLegend,#400522)
Validation	All antibodies have been used according to manufacturer's instructions. All antibodies were vallidated on either mouse or human tissue sections. For details of verification, relevant citations or further information see the manufacturer's websites.

Animals and other research organisms

Policy information about studies involving animals; ARRIVE guidelines recommended for reporting animal research, and Sex and Gender in Research

Laboratory animals	All mice were bred and maintained under specific pathogen-free conditions in the bioresource facilities of the La Trobe University or Austin Health. All strains were maintained on a 12-hour light/dark cycle at constant temperature. Co-housed, age- and gendermatched<br>littermates were utilized for all experiments. All interventions were performed during the light cycle on both male and female mice. All animals had free access to water and food (standard chow).<br>gp130+/+, 16-week-old mice<br>gp130F/F, 16-week-old mice<br>BAC(Dclk1::CreERT2);R5-IL5dtTomato-IRESCre;LSLRosa26DTA/+, 60-week-old mice<br>BAC(Dclk1::CreERT2);Rosa26DTA/+, 16-week-old mice<br>R5-IL5+/+;LSLRosa26DTA/+, 60-week-old mice<br>gp130F/F; BAC(Dclk1::CreERT2);Rosa26DTA/+, 16-week-old mice<br>gp130F/F;Rosa26DTA/+, 16-week-old mice<br>gp130F/F;R5-IL5dtTomato-IRESCre;LSLRosa26DTA/+, 16-week-old mice<br>gp130F/F;R5-IL5+/+;LSLRosa26DTA/+, 16-week-old mice
Wild animals	Wild animals are not involved in this study.
Reporting on sex	Mice used were from both male and female sexes. Final analysis does not include data on sex as there was not correlation between sex and outcome in our experiments.
Field-collected samples	Field-collected samples are not involved in this study.
Ethics oversight	All animal studies were approved by the Animal Ethics Committee of Austin Health (A2019_05602, A2015_05289) or La Trobe University (AEC 17-73).

Note that full information on the approval of the study protocol must also be provided in the manuscript.

Flow Cytometry

Plots

Confirm that:

☒ The axis labels state the marker and fluorochrome used (e.g. CD4-FITC).

☒ The axis scales are clearly visible. Include numbers along axes only for bottom left plot of group (a 'group' is an analysis of identical markers).

☒ All plots are contour plots with outliers or pseudocolor plots.

☒ A numerical value for number of cells or percentage (with statistics) is provided.

Methodology

Sample preparation	Tissues were cut into 1 mm pieces and digested in collagenase/dispase (Roche) and DNase I (Roche) in Ca2+- and Mg2+-freeHanks medium plus 5% FCS for 30 minutes at 37°C with gentle shaking. Samples were then vortexed for 15 seconds, filtered and washed in PBS plus 5% FCS. Single cell suspensions were blocked with FC block (Invitrogen) for 20 minutes at 4C, before staining with fluorophore-conjugated primary antibodies for 20 minutes at 4oC in the dark. Cells were washed twice and re-suspended in PBS supplemented with 5% FCS prior to analysis.
Instrument	Aria III cell sorter and BD FACS Canto
Software	Data was analyzed with FlowJo (Version 10)
Cell population abundance	All samples came from gastric tissue of mice, with the stomach being surgically separated from the mice before processing. As such, all cells were gastric cells. Tuft cells accounted for between 1-10% of all live EpCAM cells, while ILC2s accounted for between 0.1-4% or all live CD45.2 cells.
Gating strategy	Isotype antibodies and fluorescent-minus-one (FMO) controls were used to estimate background fluorescence in combination with either compensation beads and/or unstained controls. Dead cells were detected and excluded from analysis using Sytox Blue or Fixable Viability Dye, eF506. Tuft cells were identified as EpCAM+CD45-/lowCD24+SiglecF +.Inflammatory ILC2s were identified as ST2-KLRG1+CD90.2+Lineage-(CD11b-CD11c-CD19-Ly-6G-NK1.1-CD3-)CD45+. NaturalILC2s were identified as ST2+KLRG1+CD90.2+Lineage-CD45+.

☒ Tick this box to confirm that a figure exemplifying the gating strategy is provided in the Supplementary Information.
